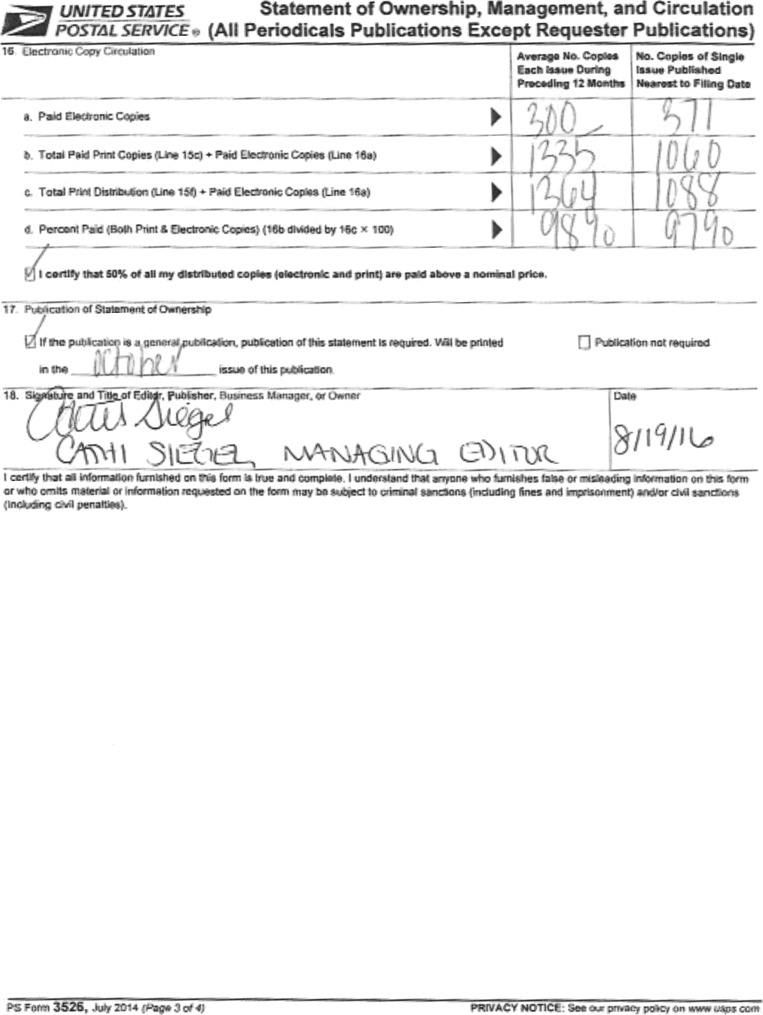# Statement of Ownership, Management, and Circulation

**DOI:** 10.4269/ajtmh.954soo

**Published:** 2016-10-05

**Figure 1:**
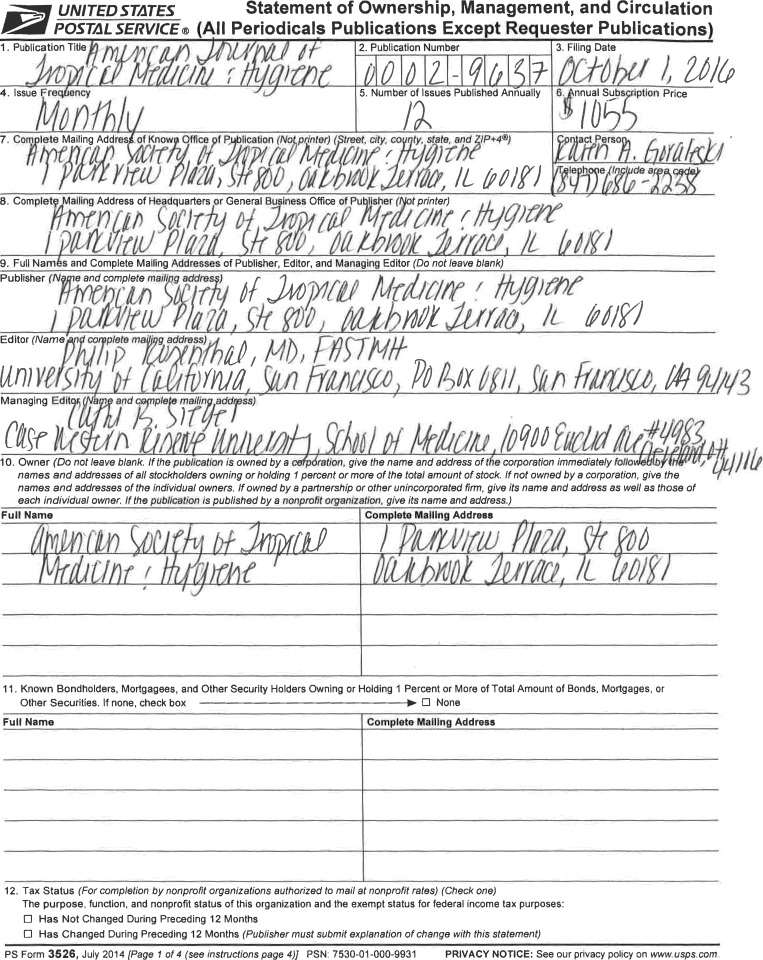

**Figure 2:**
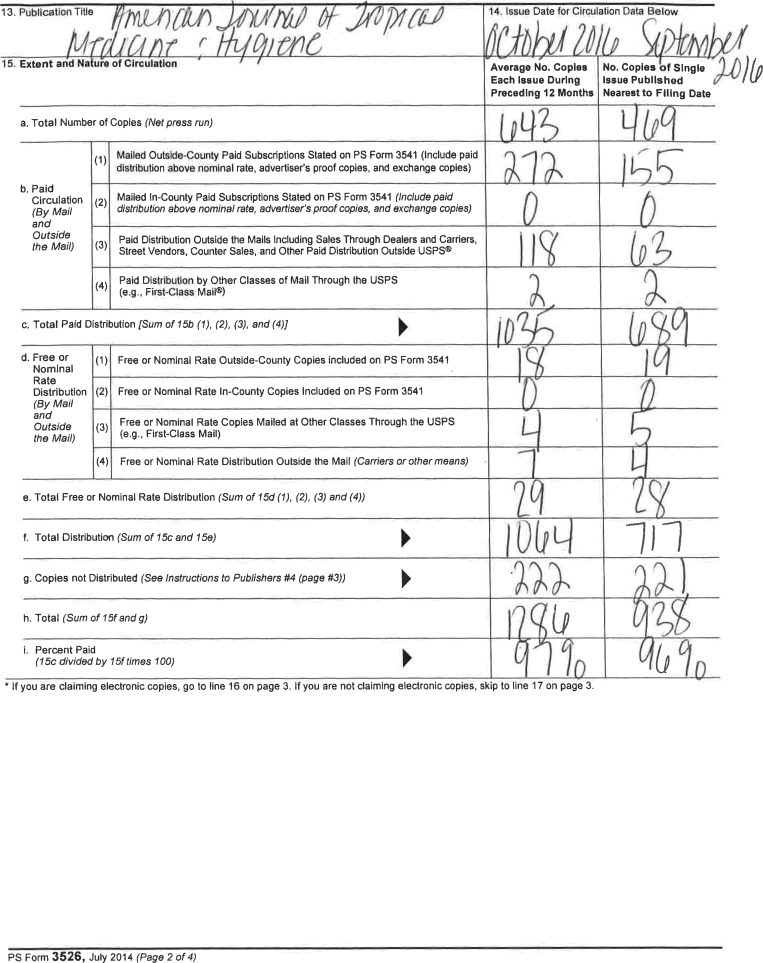

**Figure 3:**